# Supplementary material for: Topographic signatures of global object perception in human visual cortex
Source: Neuroimage. 2020 Oct 15;220:116926. doi: 10.1016/j.neuroimage.2020.116926 (PMC7573540; doi:10.1016/j.neuroimage.2020.116926)
Supplement: Supplementary Methods and Results [file mmc1.pdf]

Topographic Signatures of Global Object Perception in Human Visual Cortex

Susanne Stoll[a,*], Nonie J. Finlayson[a,1], D. Samuel Schwarzkopf[a,2]

[a]*Experimental Psychology, University College London, 26 Bedford Way, London, WC1H 0AP, UK*

Supplementary material

1. Supplementary methods and results

1.1. Diamond experiment

1.1.1. Data analysis

Perceptual durations. Participants' key presses were used to calculate the durations of the diamond and no-diamond percept. If the same key was pressed multiple times in succession, the resulting subdurations were summed up. The period from the onset of the diamond display until participants' first key press was discarded. For each participant and the data pooled across participants, we then fit the durations for the diamond and no-diamond percept with a two-parameter (α: shape, β: rate) gamma probability density function using the maximum likelihood method. The resulting fits were superimposed onto a density histogram of the perceptual durations (bin width: 2 s).

Eye position. To examine eye position stability, we analyzed the vertical and horizontal position samples corresponding to the durations of all perceptual intervals (i.e., diamond, no-diamond, and fixation). As for the analysis of perceptual durations (see Supplementary material 1.1.1 Data analysis), the interval from the onset of the diamond display until the first key press was discarded. Moreover, horizontal and vertical position samples with unavailable position information (e.g., when participants blinked or the signal dropped) were removed. This procedure resulted in substantial data loss for P4 ($>$ 20 % of all samples in any perceptual interval in $>$ 20 % of all runs rounded up to the next integer). We therefore excluded P4 from all subsequent analyses.

Slow drifts in the eye tracking signal were counteracted by detrending the time series of horizontal and vertical posites in each run using a robust time-windowed filter. To this end, we passed a sliding window (length: 10 s) through each time series by translating its midpoint from one time point to the next (range: 5-430 s; step size: 5 s) and calculating the median over all position samples within the current window. Note that all sliding windows were right-bounded except for the last one which was right-open to account for slight measurement imprecision. The resulting window-wise medians were then subtracted from the corresponding eye position samples and very extreme values ($> |8.5$ dva$|$ in both the horizontal and vertical direction; cut-off determined based on screen height) eliminated afterwards.

To explore percept-specific differences in the bivariate distribution of position samples, we generated *bagplots* (Rousseeuw et al., 1999) across all runs for each perceptual interval and participant. Bagplots represents a (model-free) 2D generalization of univariate boxplots and typically include a *center* point corresponding to the depth median, a *bag* demarking a region containing 50% of the data, a *fence* (usually not visualized) generated via inflating the bag by a factor of 3, and a *loop* circumscribing data points between the bag and the fence. Data points beyond the fence are considered outliers.

To further asses fixation variability, we determined the run-wise robust scale parameter S_n (Rousseeuw & Croux, 1993) for the horizontal and vertical position samples of each perceptual interval. Moreover, to check for systematic biases towards the (perceived) movement direction of the diamond stimulus more directly, we subtracted the S_n of the vertical samples from the S_n of the horizontal samples for each interval type (fixation served as a benchmark here). If participants' gaze was indeed biased towards the horizontal direction during the diamond precept and the vertical direction during the no-diamond precept, we should observe a positive difference for the diamond inter-

*Corresponding author

Email address: stollsus@gmail.com (Susanne Stoll)

[1]Ipsos, Suite 3a, 201 Leichhardt Street Spring Hill, Brisbane, Queensland, 4000, Australia

[2]School of Optometry and Vision Science, The University of Auckland, 85 Park Road, Auckland, 1023, New Zealand

vals and a negative difference for the no-diamond intervals. Such dispersion differences should be likewise evident in the bagplots.

1.1.2. Results

Perceptual durations. The probability density histograms of the durations per perceptual state for each participant and the pooled data with superimposed gamma fit can be found in Supplementary Figure S2. Despite inter-individual variability in the shape and rate parameters, both the pooled and individual diamond and no-diamond durations seem to be well fit with a gamma distribution, suggesting they follow similar temporal dynamics. However, all participants except P2 showed a tendentially higher probability density of longer durations for the no-diamond relative to the diamond percept. Likewise, these participants showed a higher median duration for the no-diamond percept and spent a higher proportion of time in this perceptual state, which was also reflected in the pooled results. Consequently, the perception of most participants was slightly biased towards the no-diamond state.

Eye position. Supplementary Figure S3 shows bagplots of the eye position samples across runs (A.) as well as simple (B.) and differential (C.) run-wise dispersion estimates for each participant and perceptual interval. For most participants, the size of the bag was fairly small and similar across perceptual intervals, suggesting a small degree of position variability unrelated to the perceptual interval at hand. Moreover, for most participants, the shape of the bag and loop was largely symmetric (circular or elliptical) and comparable across perceptual intervals, speaking against a systematic skew. For P3, however, the size of the bag tended to be generally larger compared to other participants and stretched out horizontally in the no-diamond relative to the diamond interval, highlighting greater position variability and a modest horizontal bias (see all Supplementary Figure S3, A.). Importantly, however, this bias ran counter to the perceived vertical movement during the no-diamond percept. The run-wise dispersion estimates (Supplementary Figure S3, B. and C.) confirmed all these observations whilst additionally revealing that the horizontal bias for P3 was predominantly present in the last run.

1.2. Dots experiment

1.2.1. Data analysis

Eye position. The eye position analysis was conducted as in the diamond experiment. However, due to different design parameters, the range of the sliding window midpoints was 5-355 s here. Moreover, as a result of signal drop due to technical issues, the data for P3 and P4 were removed from all analyses.

1.2.2. Results

Eye position. Supplementary Figure S7 lists the bagplots of the eye position samples across runs per participant and perceptual interval (A.) together with the corresponding run-wise simple (B.) and differential (C.) dispersion estimates. For all participants and perceptual intervals, the shape of the bag and loop was reasonably symmetric and the size of the bag fairly small. Importantly, these characteristics were comparable across perceptual intervals (see all Supplementary Figure S7, A.). The run-wise dispersion estimates corroborated theses observations (Supplementary Figure S7 B. and C.). Collectively, these results suggests little position variability and no systematic skew related to the motion directions of the dots stimulus.

1.3. Dots quadrant experiment

1.3.1. Data analysis

Eye position. The eye position analysis was conducted as in the diamond and dots experiment except that the range of the sliding window midpoints amounted to 5-370 s here (since we also collected eye data during the final blank, see 5.1.4 Procedure).

1.3.2. Results

Eye position. Supplementary Figure S11 depicts bagplots for the eye position samples of all runs (A.) along with run-wise simple (B.) and differential (C.) dispersion estimates per participant and perceptual state. For all participants, we observed relatively small-sized bags as well as symmetrically-shaped loops and bags that were highly similar across perceptual intervals (see all Supplementary Figure S11, A.). The run-wise dispersion estimates confirmed this pattern (Supplementary Figure S11, B. and C.). These outcomes suggest an overall low position variability that seems unrelated to the motion direction in the horizontal or vertical condition.

2. Supplementary figures and tables

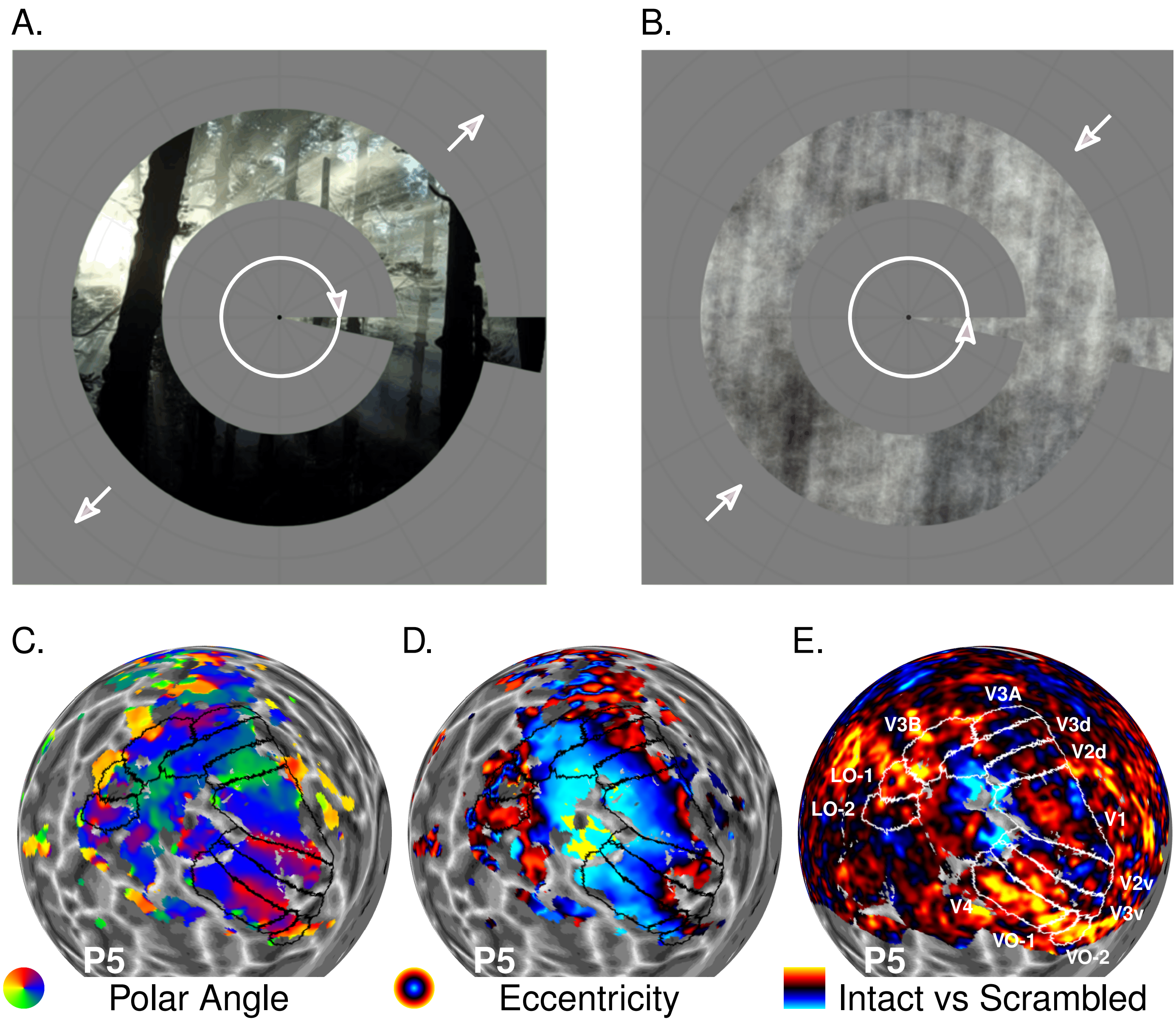

Figure S1. **Retinotopic mapping experiment**| Example frames of the wedge-and-ring stimulus and smooth cortical maps of P5's left hemisphere projected onto a spherical surface model. **A.** Intact, colorful carrier pattern. **B.** Phase-scrambled version of the carrier pattern in A. The white arrows indicate the movement direction of the wedge-and-ring aperture, which was either clockwise and expanding (A.), counterclockwise and contracting (B.), clockwise and contracting, or counterclockwise and expanding (not shown, respectively). **C.** Polar angle map. **D.** Eccentricity map. Vertices with a pRF surpassing an eccentricity of 15 dva were discarded (no other post-smoothing thresholding was applied). Note that these pRF maps were subjected to the experiment-specific smoothing procedure in the diamond experiment (see 3.1.7 Data analysis). The color disks represent the color schemes used to label different visual field portions. **E.** Differential brain activity resulting from contrasting periods of intact to periods of phase-scrambled images. Differential betas surpassing a value of $\pm$ 2 were set to that value. Cold colors reflect negative and warm colors positive differential beta values as indicated by the color bar. White or black lines denote the boundaries between visual areas. The gray scale pattern of the surface model reflects the cortical curvature. Darker regions depict sulci and lighter regions gyri. P5 = Participant 5. VO = Ventral-occipital area. LO = Lateral-occipital area. PRF = Population receptive field.

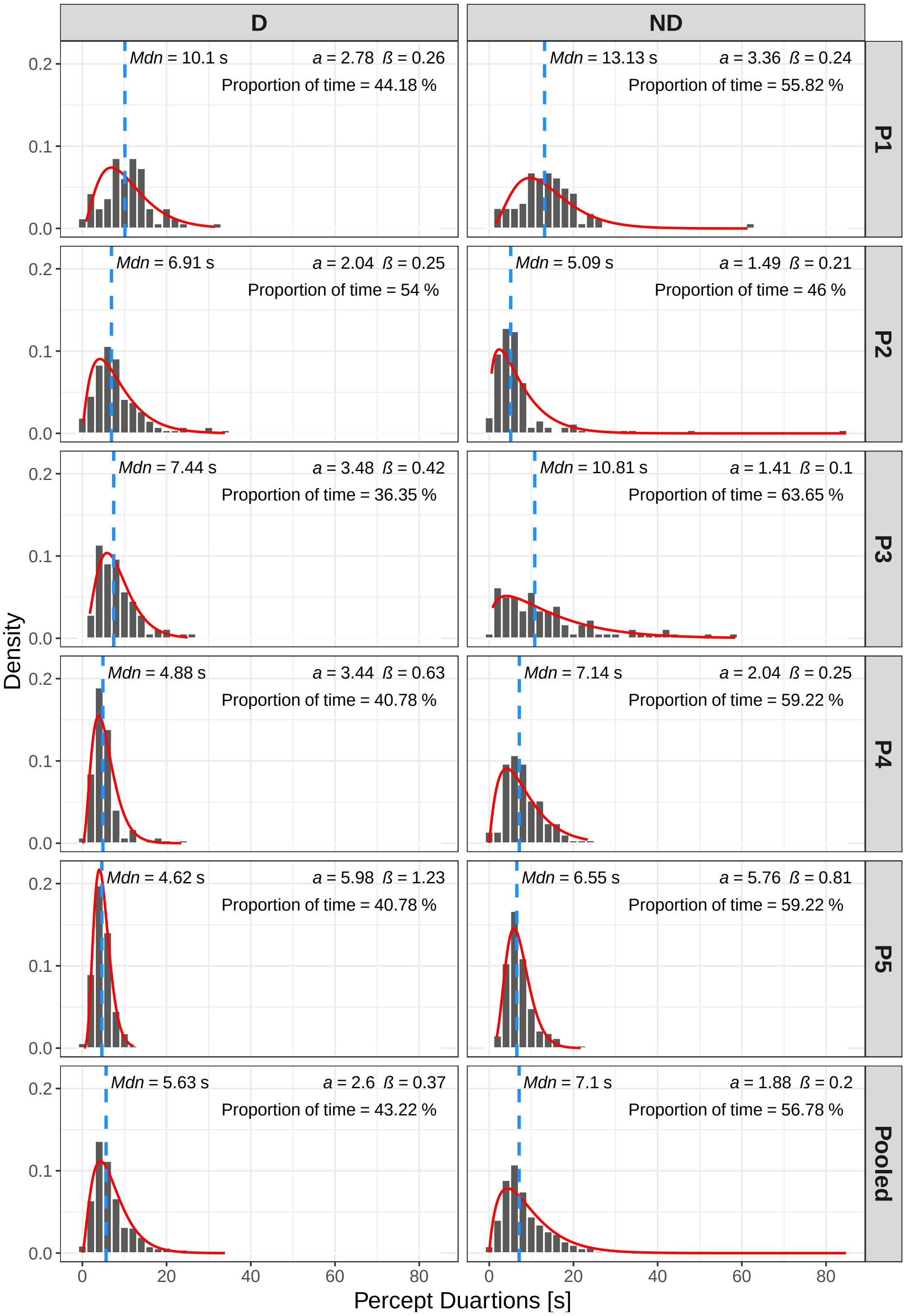

Figure S2. **Diamond experiment**| Probability density histograms of the durations corresponding to the diamond and no-diamond percept with superimposed gamma functions. The red line depicts the fitted gamma curve and the blue line the median duration. α, β = Shape and rate parameter of the gamma distribution, respectively. Proportion of time = Proportion of time spent in the respective perceptual state. D = Global, diamond percept. ND = Local, no-diamond percept. P1-P5 = Participant 1-5. Pooled = Data pooled across all 5 participants.

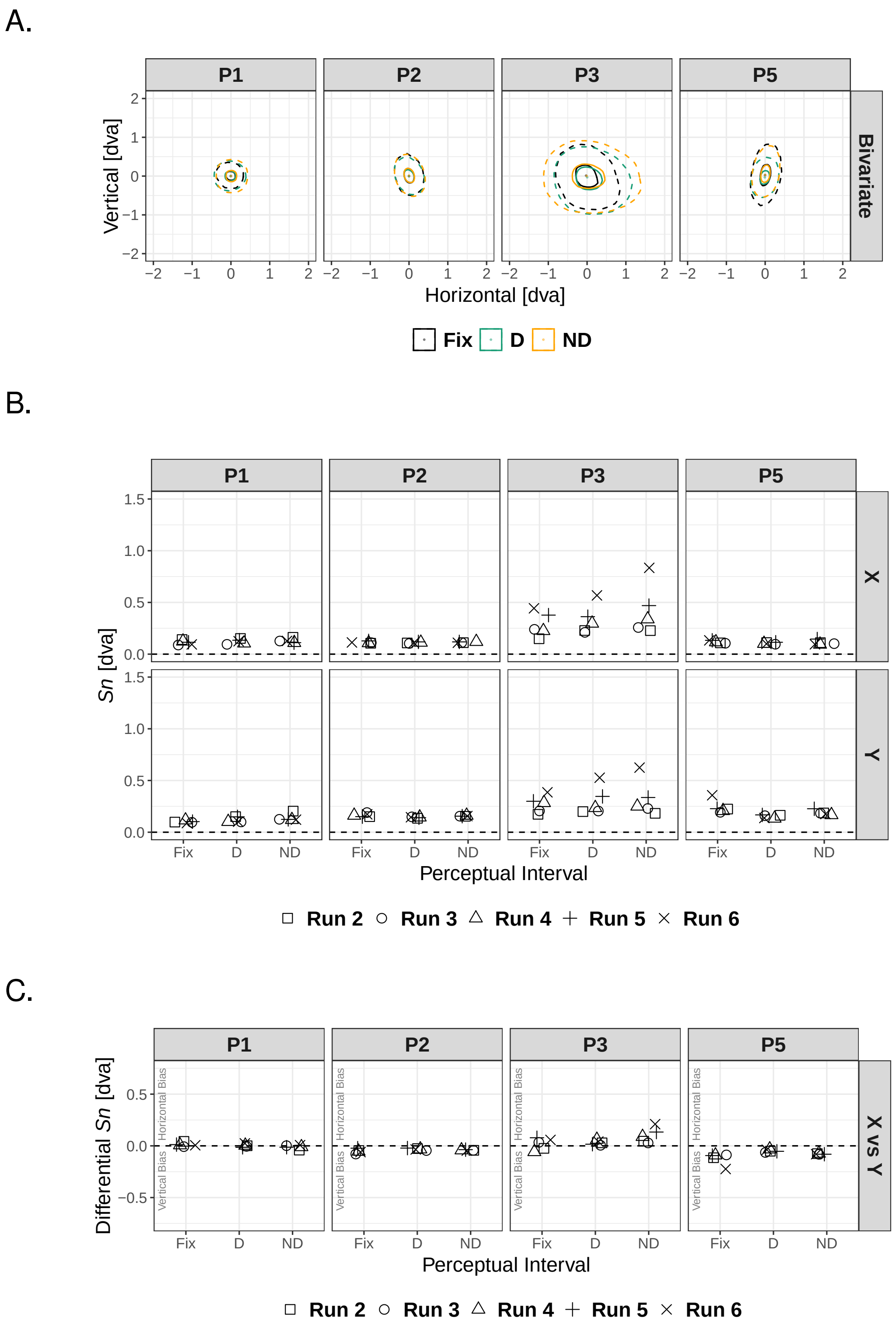

Figure S3. **Diamond experiment**| Eye position distribution and variability during the diamond and no-diamond percept and fixation intervals. **A.** Bagplots of the vertical and horizontal eye position samples across runs. Note that for reasons of visibility, only a portion of the visual field of interest is shown and outliers are dropped. **B.** Run-wise dispersion estimates for the horizontal and vertical eye position samples. **C.** Run-wise difference in dispersion estimates for the horizontal vs vertical eye position samples. Note that the location of the symbols was jittered to counteract overplotting. S_n = Robust measure of dispersion. X = Horizontal eye position samples. Y = Vertical eye position samples. Horizontal bias = Larger dispersion estimates for the horizontal vs vertical eye position samples. Vertical bias = Larger dispersion estimates for the vertical vs horizontal eye samples. D = Global, diamond percept. ND = Local, no-diamond percept. Fix = Fixation baseline. P1-P3 and P5 = Participant 1-3 and 5.

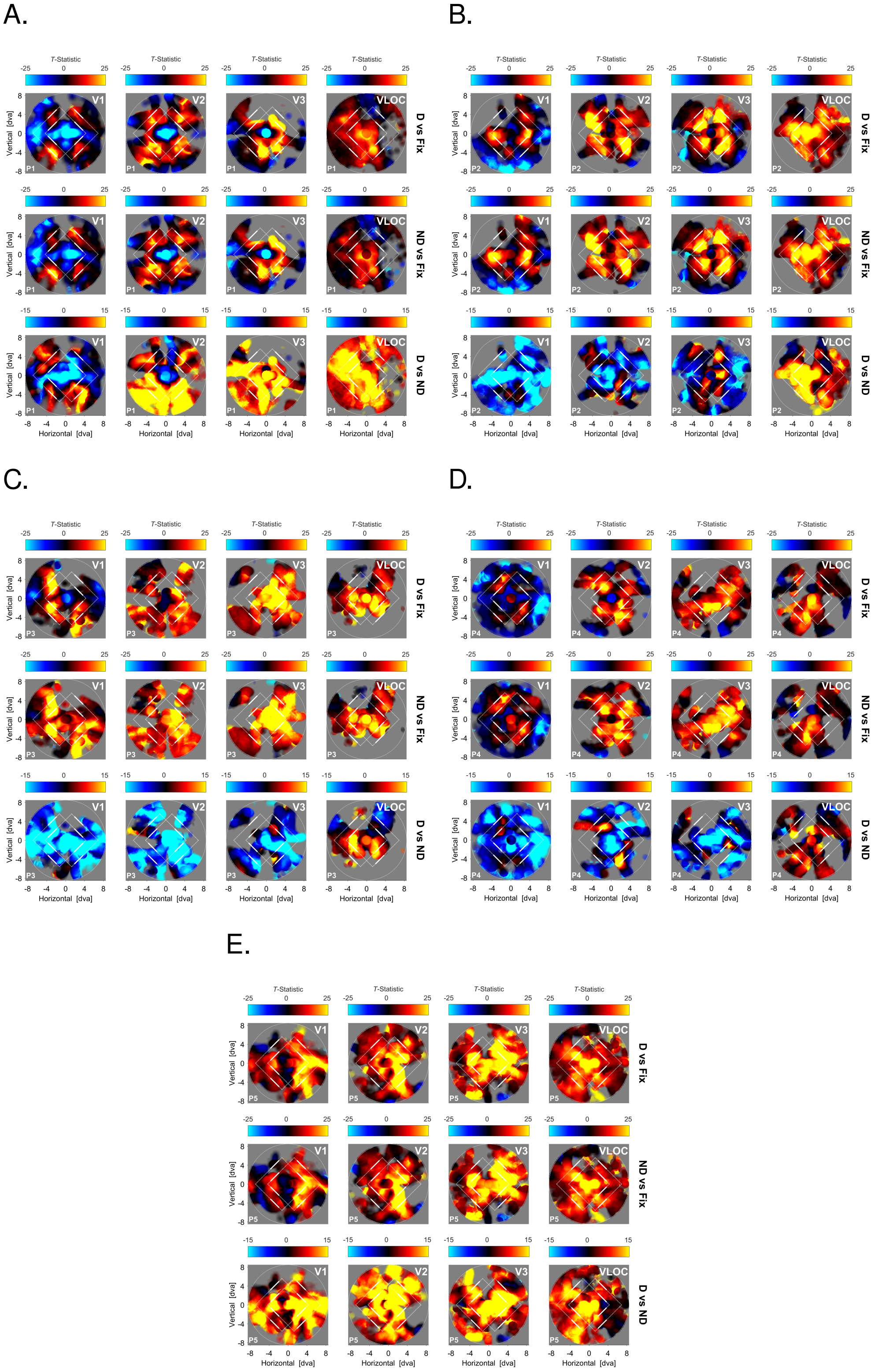

Figure S4. **Diamond experiment**| Searchlight back-projections of differential brain activity as a function of contrast of interest and visual area. **A.-E.** P1-P5. T-statistics surpassing a value of $\pm$ 25 (first and second row) or $\pm$ 15 (third row) were set to that value. The saturation of colors reflects the number of vertices with a pRF inside a given searchlight plus the inverse distance of these pRFs from the searchlight center. White lines represent the extreme positions of the diamond stimulus. White solid lines denote the visible ungrouped diamond segments. White dashed lines additionally illustrate the inferred but invisible diamond shape when the segments were grouped together. D = Global, diamond percept. ND = Local, no-diamond percept. Fix = Fixation baseline. VLOC = Ventral-and-lateral occipital complex. P1-P5 = Participant 1-5. pRF = Population receptive field.

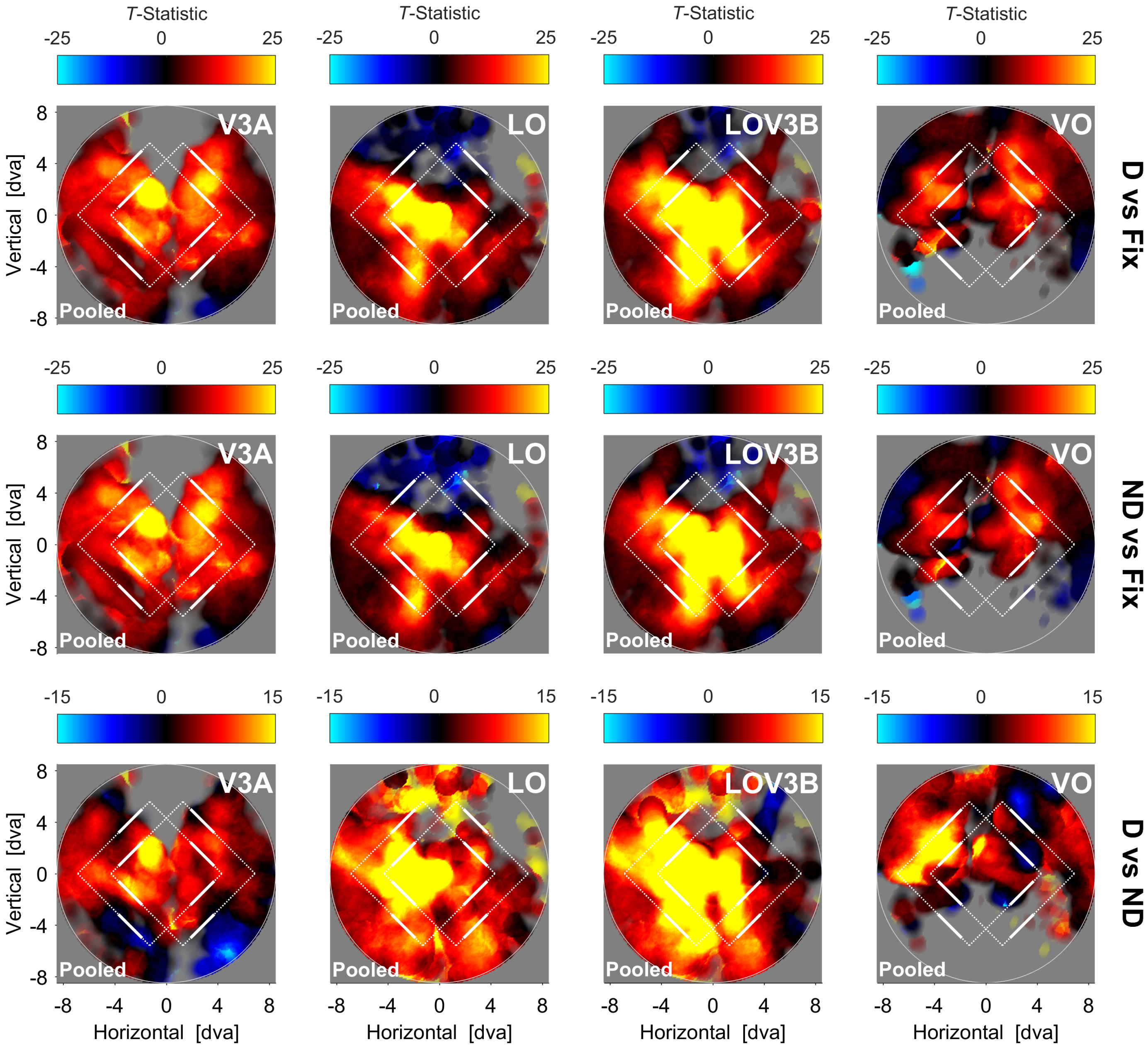

Figure S5. **Diamond experiment**| Searchlight back-projections of differential brain activity as a function of contrast of interest and visual area. Please note that the visual field coverage for these visual areas was suboptimal in some participants. T-statistics surpassing a value of $\pm$ 25 (first and second row) or $\pm$ 15 (third row) were set to that value. The saturation of colors reflects the number of vertices with a pRF inside a given searchlight plus the inverse distance of these pRFs from the searchlight center. White lines represent the extreme positions of the diamond stimulus. White solid lines denote the visible ungrouped diamond segments. White dashed lines additionally illustrate the inferred but invisible diamond shape when the segments were grouped together. D = Global, diamond percept. ND = Local, no-diamond percept. Fix = Fixation baseline. LO = Lateral-occipital area. LOV3B = Areas LO-1, LO-2, and V3B. VO = Ventral-occipital area. Pooled = Data pooled across all 5 participants. pRF = Population receptive field.

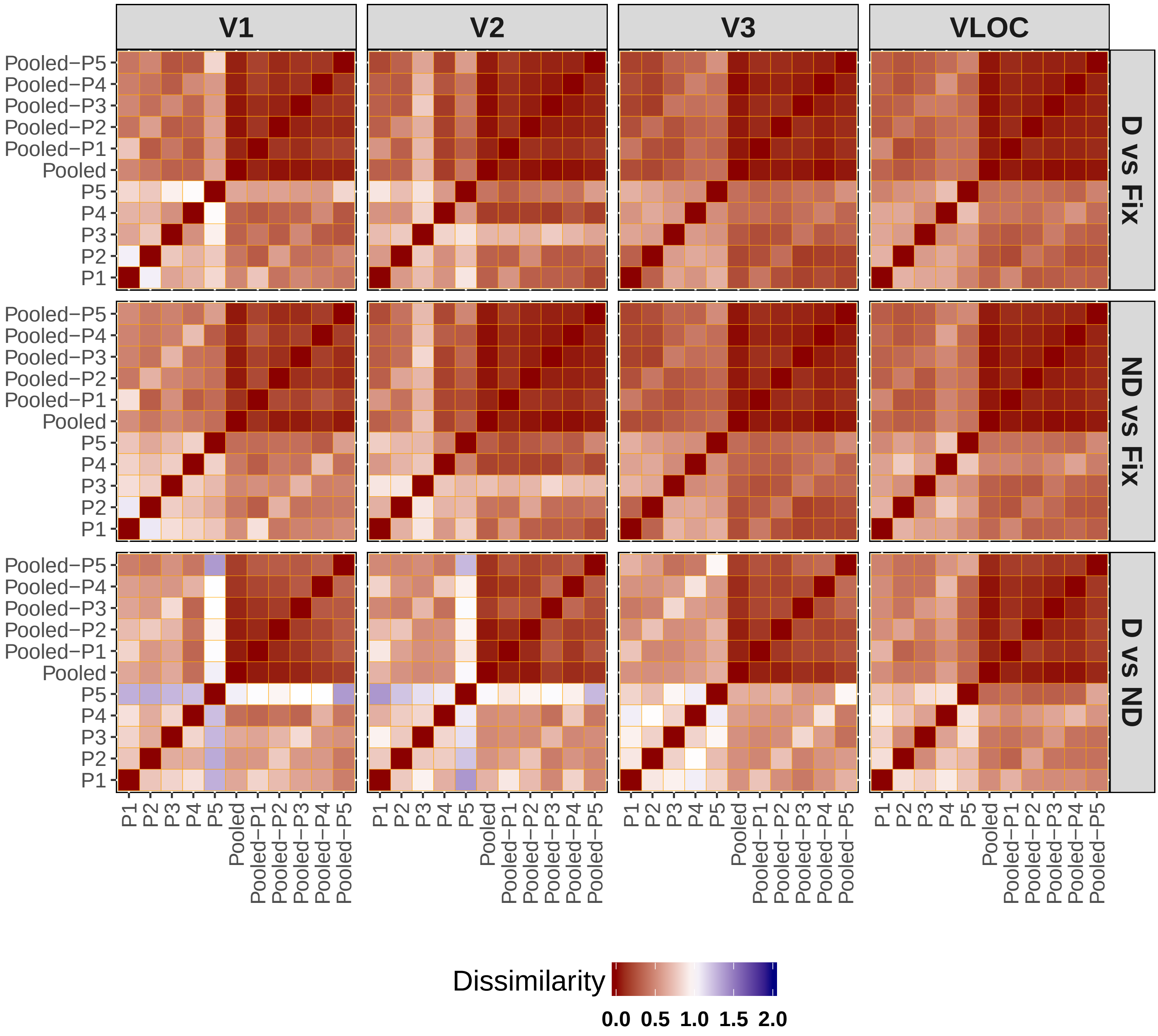

Figure S6. **Diamond experiment**| Representational dissimilarity matrices for the individual, pooled, and LOSO searchlight back-projections as a function of contrast of interest and visual area. Dissimilarities were defined as 1-Spearman correlation. D = Global, diamond percept. ND = Local, no-diamond percept. Fix = Fixation baseline. VLOC = Ventral-and-lateral occipital complex. P1-P5 = Participant 1-5. Pooled = Data pooled across all 5 participants. Pooled-P1-Pooled-P5 = Data pooled across 4 participants with 1 participant left out (as indicated by the suffix). LOSO = Leave-one-subject-out.

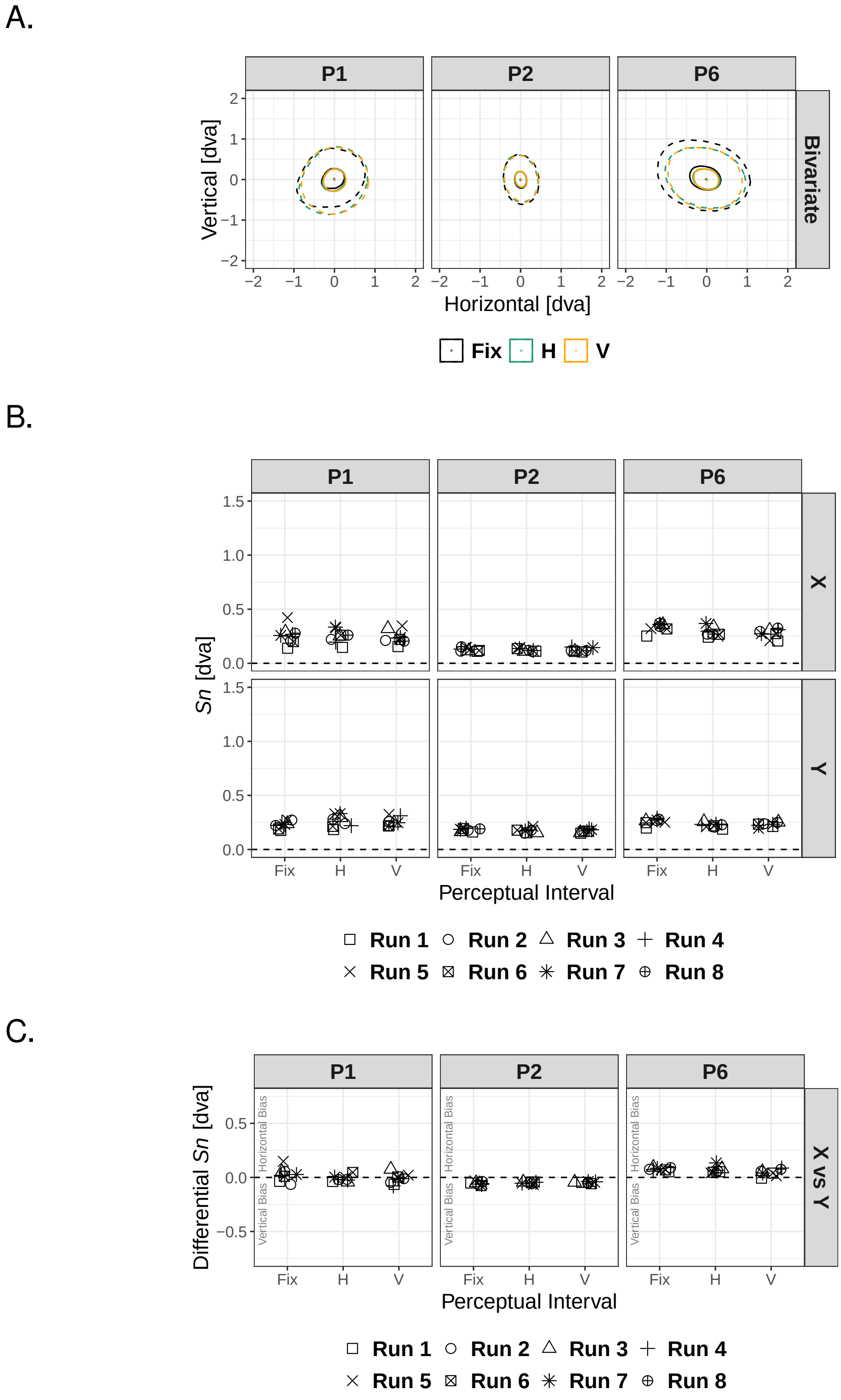

Figure S7. **Dots experiment**| Eye position distribution and variability during the horizontal and vertical condition and fixation intervals. **A.** Bagplots of the vertical and horizontal eye position samples across runs. Note that for reasons of visibility, only a portion of the visual field of interest is shown and outliers are dropped. **B.** Run-wise dispersion estimates for the horizontal and vertical eye position samples. **C.** Run-wise difference in dispersion estimates for the horizontal vs vertical eye position samples. Note that the location of the symbols was jittered to counteract overplotting. S_n = Robust measure of dispersion. X = Horizontal eye position samples. Y = Vertical eye position samples. Horizontal bias = Larger dispersion estimates for the horizontal vs vertical eye position samples. Vertical bias = Larger dispersion estimates for the vertical vs horizontal eye samples. H = Global, horizontal condition. V = Local, vertical condition. Fix = Fixation baseline. P1-P2 and P6 = Participant 1-2 and 6.

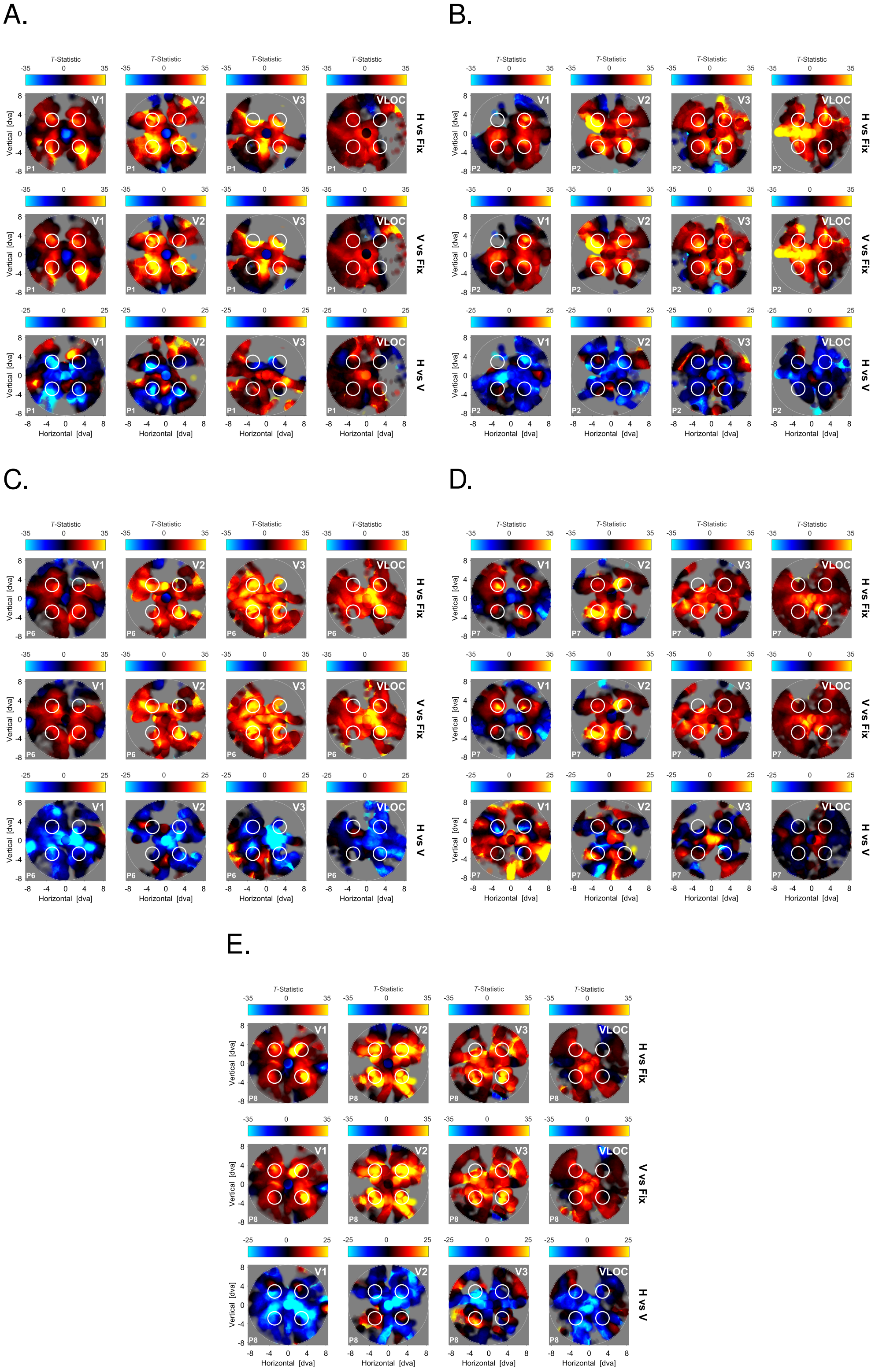

Figure S8. **Dots experiment**| Searchlight back-projections of differential brain activity as a function of contrast of interest and visual area. **A.-E.** P1-P2 and P6-P8. T-statistics surpassing a value of $\pm$ 35 (first and second row) or $\pm$ 25 (third row) were set to that value. The saturation of colors reflects the number of vertices with a pRF inside a given searchlight plus the inverse distance of these pRFs from the searchlight center. White lines represent the spatial extent of the circular apertures carrying the RDK. H = Global, horizontal condition. V = Local, vertical condition. Fix = Fixation baseline. VLOC = Ventral-and-lateral occipital complex. P1-P2 and P6-P8 = Participant 1-2 and 6-8. RDK = Random dot kinematogram. pRF = Population receptive field.

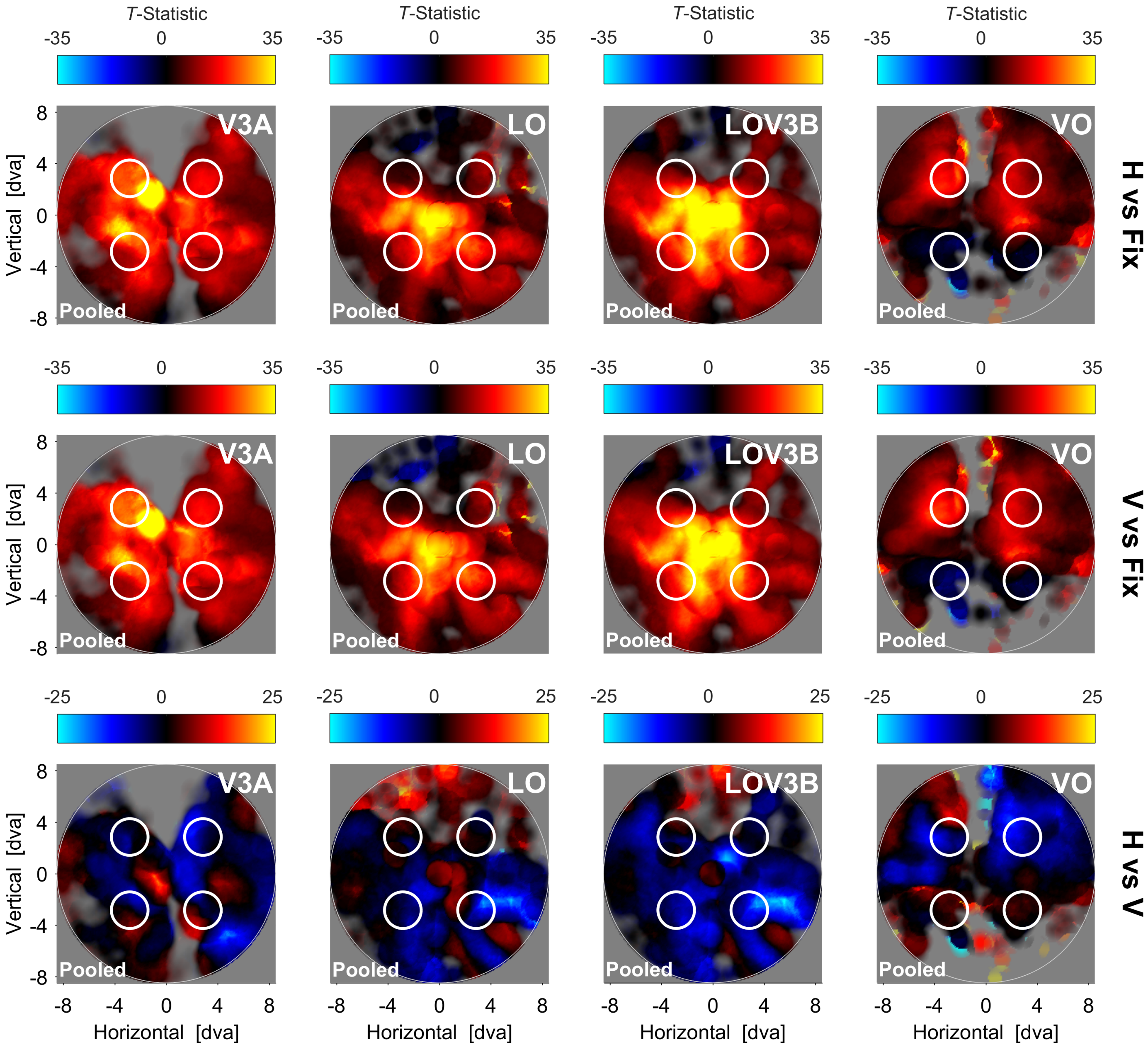

Figure S9. **Dots experiment**| Searchlight back-projections of differential brain activity as a function of contrast of interest and visual area. Please note that the visual field coverage for these visual areas was suboptimal in some participants. T-statistics surpassing a value of ± 35 (first and second row) or ± 25 (third row) were set to that value. The saturation of colors reflects the number of vertices with a pRF inside a given searchlight plus the inverse distance of these pRFs from the searchlight center. White lines represent the spatial extent of the circular apertures carrying the RDK. H = Global, horizontal condition. V = Local, vertical condition. Fix = Fixation baseline. LO = Lateral-occipital area. LOV3B = Areas LO-1, LO-2, and V3B. VO = Ventral-occipital area. Pooled = Data pooled across all 5 participants. RDK = Random dot kinematogram. pRF = Population receptive field.

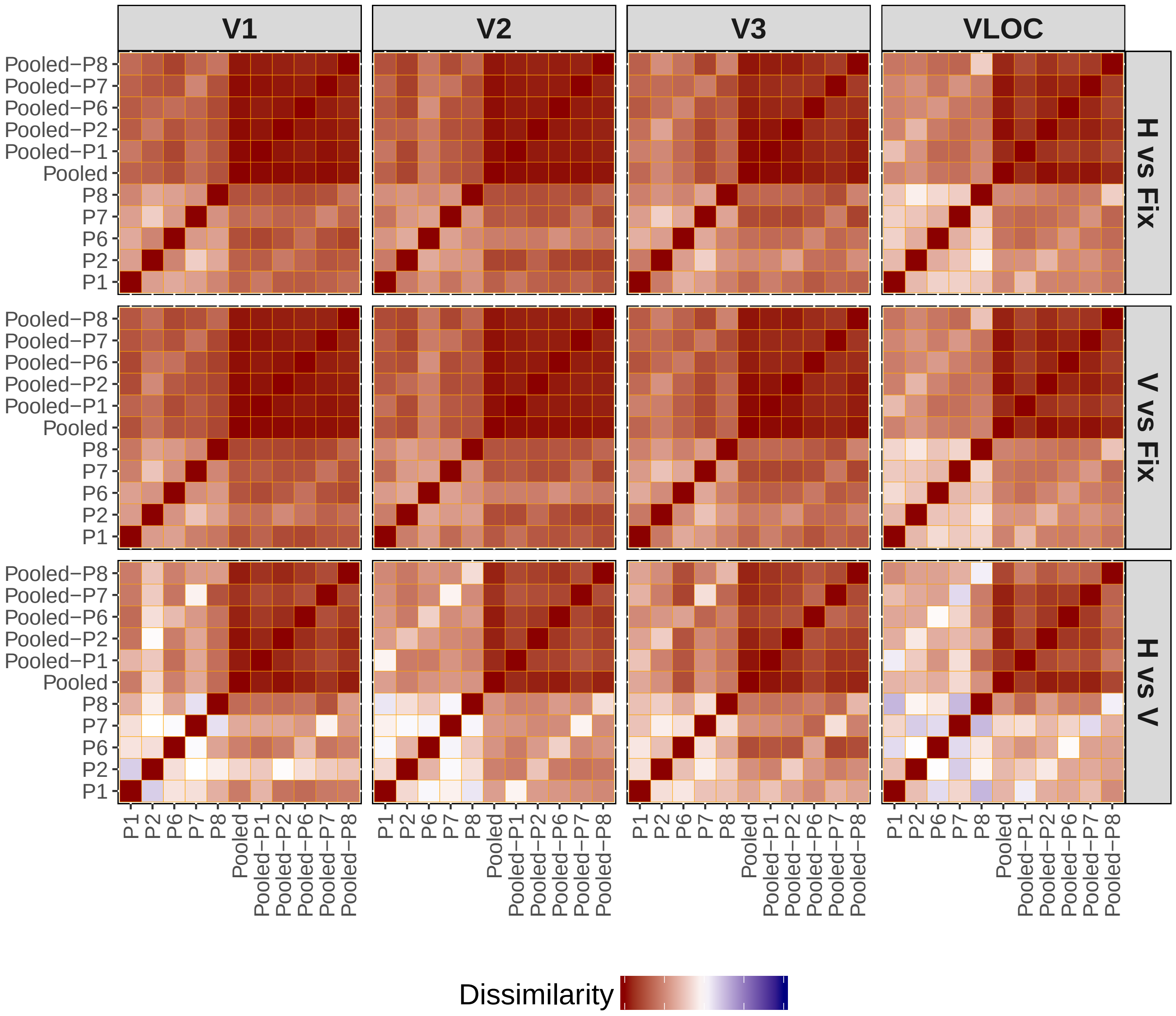

Figure S10. **Dots experiment**| Representational dissimilarity matrices for the individual, pooled, and LOSO searchlight back-projections as a function of contrast of interest and visual area. Dissimilarities were defined as 1-Spearman correlation. H = Global, horizontal condition. V = Local, vertical condition. Fix = Fixation baseline. VLOC = Ventral-and-lateral occipital complex. P1-P2 and P6-P8 = Participant 1-2 and 6-8. Pooled = Data pooled across all 5 participants. Pooled-P1-Pooled-P2 and Pooled-P6-Pooled-P8 = Data pooled across 4 participants with 1 participant left out (as indicated by the suffix). LOSO = Leave-one-subject-out.

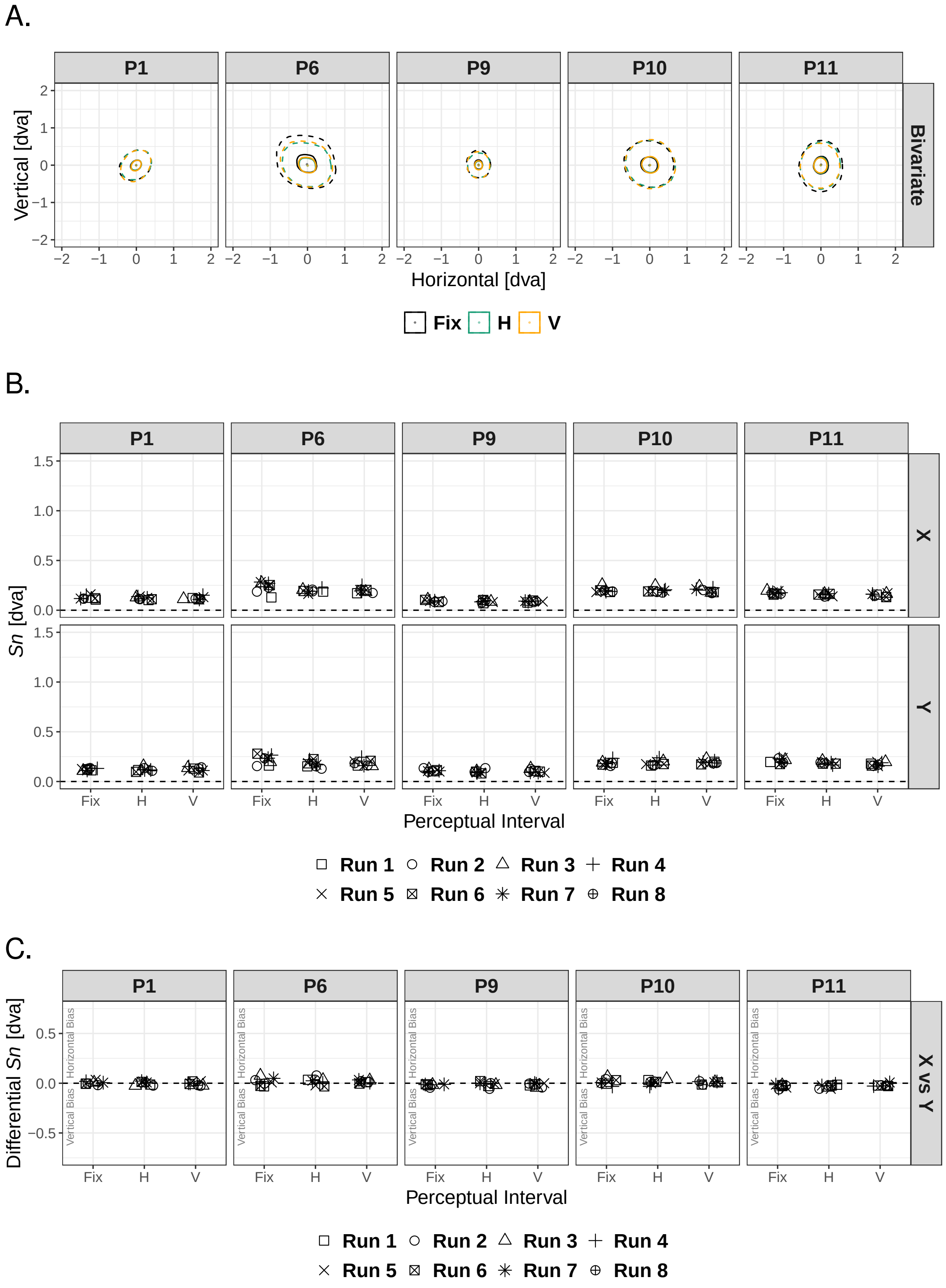

Figure S11. **Dots quadrant experiment**| Eye position distribution and variability during the horizontal and vertical condition and fixation intervals. **A.** Bagplots of the vertical and horizontal eye position samples across runs. Note that for reasons of visibility, only a portion of the visual field of interest is shown and outliers are dropped. **B.** Run-wise dispersion estimates for the horizontal and vertical eye position samples. **C.** Run-wise difference in dispersion estimates for the horizontal vs vertical eye position samples. Note that the location of the symbols was jittered to counteract overplotting. S_n = Robust measure of dispersion. X = Horizontal eye position samples. Y = Vertical eye position samples. Horizontal bias = Larger dispersion estimates for the horizontal vs vertical eye position samples. Vertical bias = Larger dispersion estimates for the vertical vs horizontal eye samples. H = Global, horizontal condition. V = Local, vertical condition. Fix = Fixation baseline. P1, P6, and P9-11 = Participant 1, 6, and 9-11.

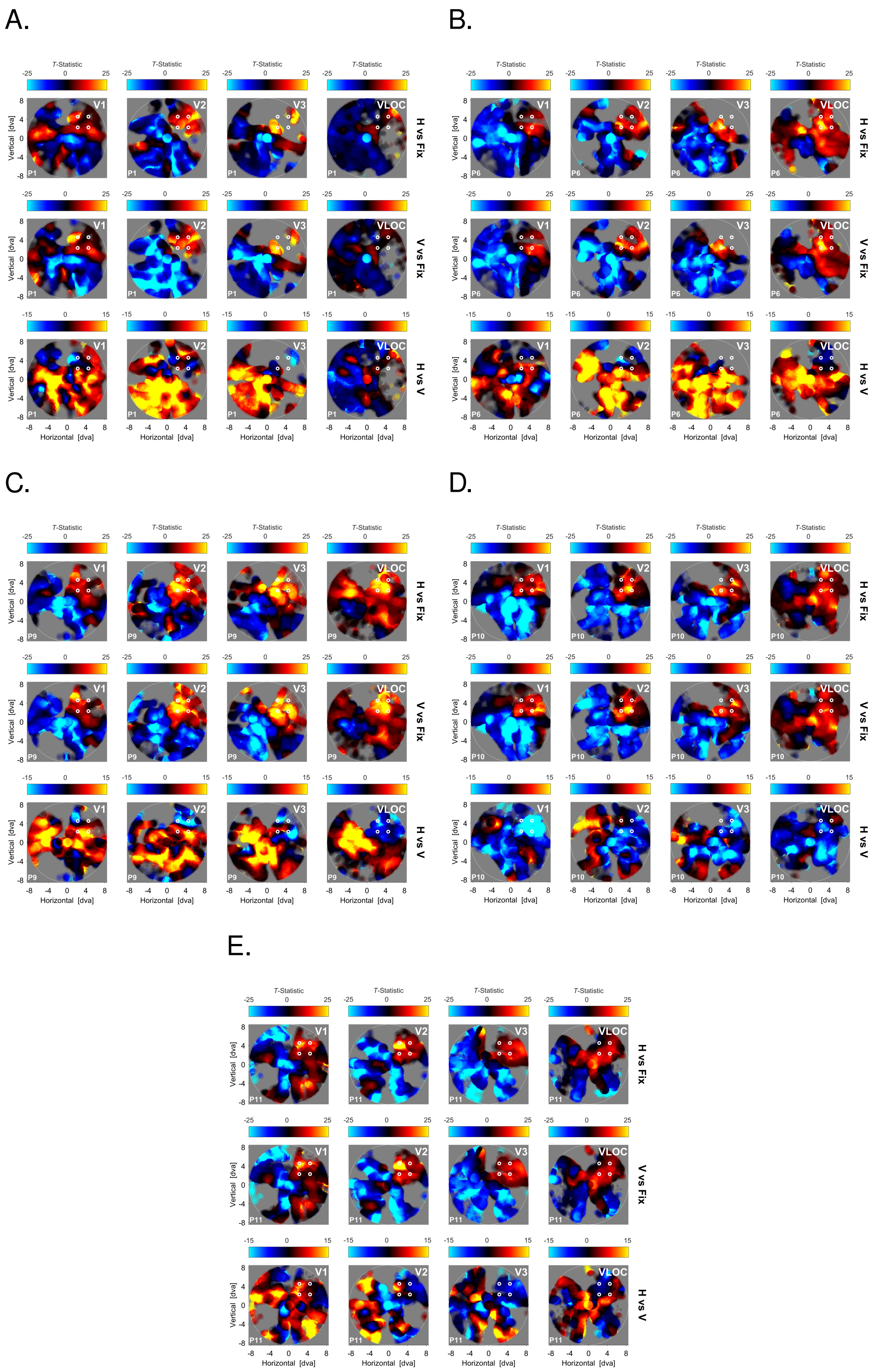

Figure S12. **Dots quadrant experiment**| Searchlight back-projections of differential brain activity as a function of contrast of interest and visual area. **A.-E.** P1, P6, P9-P11. T-statistics surpassing a value of $\pm$ 25 (first and second row) or $\pm$ 15 (third row) were set to that value. The saturation of colors reflects the number of vertices with a pRF inside a given searchlight plus the inverse distance of these pRFs from the searchlight center. White lines represent the spatial extent of the circular apertures carrying the RDK. H = Global, horizontal condition. V = Local, vertical condition. Fix = Fixation baseline. VLOC = Ventral-and-lateral occipital complex. P1, P6, and P9-P11 = Participant 1, 6, and 9-11. RDK = Random dot kinematogram. pRF = Population receptive field.

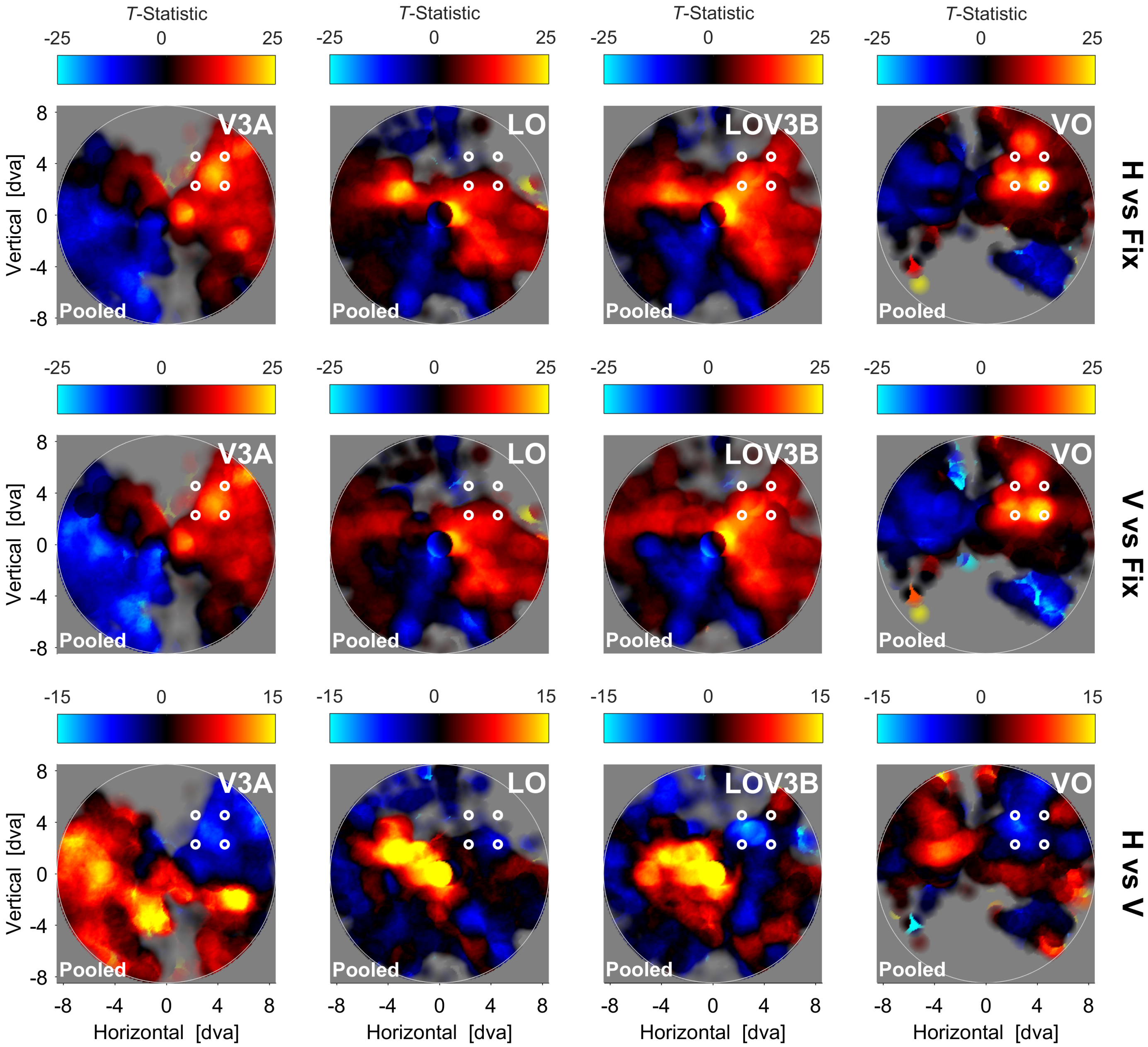

Figure S13. **Dots quadrant experiment**| Searchlight back-projections of differential brain activity as a function of contrast of interest and visual area. Please note that the visual field coverage for these visual areas was suboptimal in some participants. T-statistics surpassing a value of $\pm$ 25 (first and second row) or $\pm$ 15 (third row) were set to that value. The saturation of colors reflects the number of vertices with a pRF inside a given searchlight plus the inverse distance of these pRFs from the searchlight center. White lines represent the spatial extent of the circular apertures carrying the RDK. H = Global, horizontal condition. V = Local, vertical condition. Fix = Fixation baseline. LO = Lateral-occipital area. LOV3B = Areas LO-1, LO-2, and V3B. VO = Ventral-occipital area. Pooled = Data pooled across all 5 participants. RDK = Random dot kinematogram. pRF = Population receptive field.

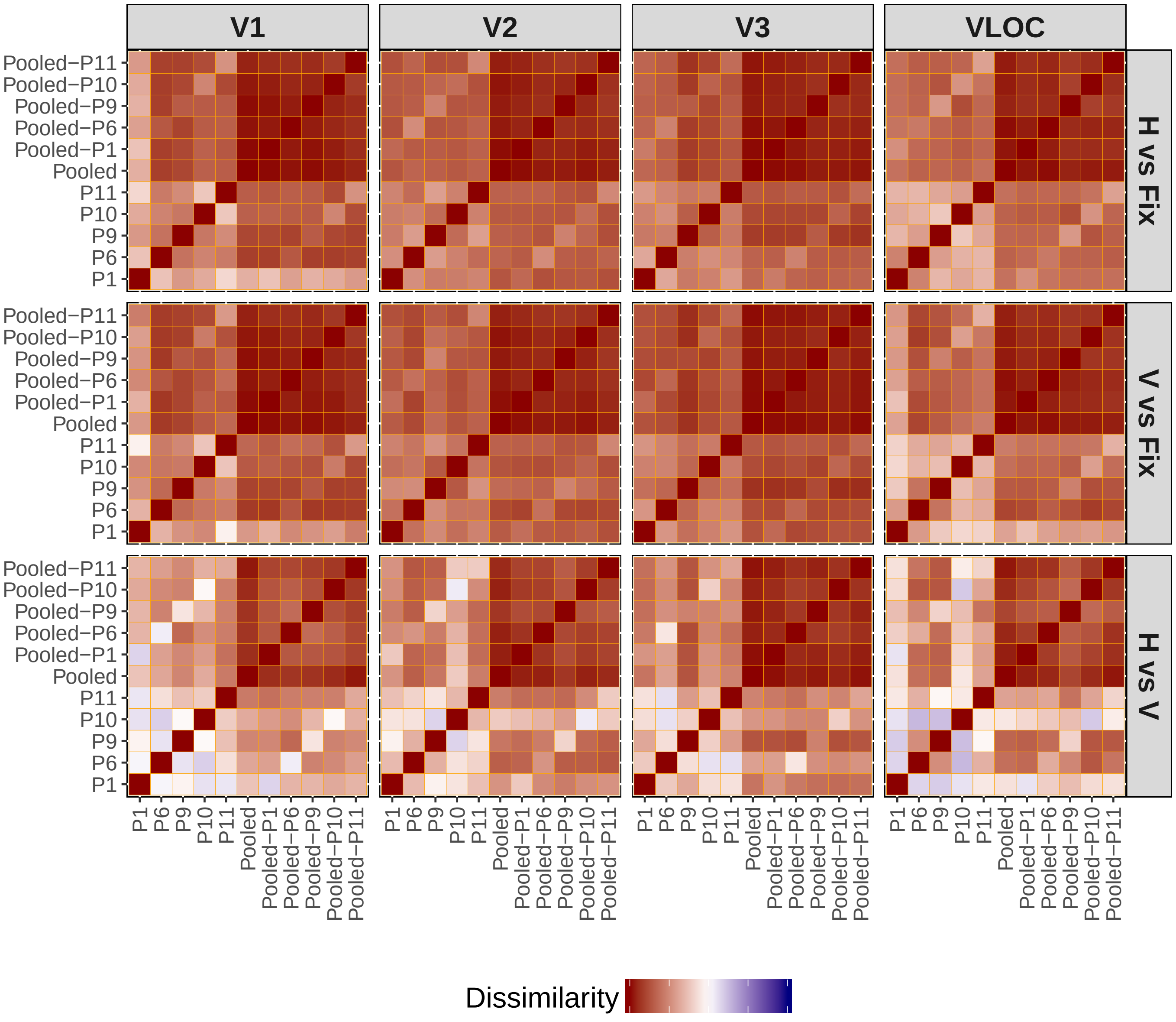

Figure S14. **Dots quadrant experiment**| Representational dissimilarity matrices for the individual, pooled, and LOSO searchlight back-projections as a function of contrast of interest and visual area. Dissimilarities were defined as 1-Spearman correlation. H = Global, horizontal condition. V = Local, vertical condition. Fix = Fixation baseline. VLOC = Ventral-and-lateral occipital complex. P1, P6, and P9-P11 = Participant 1, 6, and 9-11. Pooled = Data pooled across all 5 participants. Pooled-P1, Pooled-P6, and Pooled-P9-Pooled-P11 = Data pooled across 4 participants with 1 participant left out (as indicated by the suffix). LOSO = Leave-one-subject-out.

Table S1
Software Environments and Toolboxes

Implemented procedures	Software environment (version)	Toolbox (version)
Experiments	Matlab R2014a (8.3)[1]	PTB (3.0.11)[4]
Preprocessing		
Realignment/unwarping/coregistration	Matlab R2016b (9.1)[1]	SPM8 (6313; default parameters)[5]
Surface reconstruction	FreeSurfer (5.3.0)[2]	
Surface projection/detrending/standardization	Matlab R2016b (9.1)[1]	SamSrf (5.84)[6]
		SPM8 (6313)[5]
pRF estimation	Matlab R2016b (9.1)[1]	SamSrf (5.84)[6]
		SPM8 (6313)[5]
Delineations	Matlab R2016b (9.1)[1]	
GLM		SPM8 (6313)[5]
Surface projection/manual demarcation		SamSrf (5.84)[6]
		SPM8 (6313)[5]
Smoothing/visualizations		SamSrf (6.20)[7]
Searchlight back-projections	Matlab R2016b (9.1)[1]	
GLM		SPM8 (6313)[5]
Surface projection		SamSrf (5.84)[6]
		SPM8 (6313)[5]
Smoothing		SamSrf (6.20)[7]
Searchlight algorithm/visualization		SamSrf (6.31)[8]
Representational similarity		
Dissimilarity calculation	Matlab R2016b (9.1)[1]	
cMDS/visualizations	R (3.5.3)[3]	vegan (2.5-6)[9]
		ggplot2(3.2.1)[10]
		reshape2(1.4.3)[11]
		plyr (1.8.4)[12]
		rmatio (0.14.0)[13]
		egg (0.4.5)[14]
Perceptual durations		
Duration calculation	Matlab R2016b (9.1)[1]	
Gamma fitting/visualizations	R (3.5.3)[3]	MASS (7.3-51.4)[15]
		ggplot2 (3.2.1)[10]
		rmatio (0.14.0)[13]
		egg (0.4.5)[14]
Eye position	R (3.5.3)[3]	
Detrending/distribution/variability/visualizations		ggplot2 (3.2.1)[10]
		reshape2 (1.4.3)[11]
		plyr (1.8.4) [12]
		rmatio (0.14.0) [13]
		egg (0.4.5)[14]
		MASS (7.3-51.4)[15]
		aplpack (1.3.3)[16]
		robustbase (0.93-5)[17]

Note. [1]https://uk.mathworks.com/. [2]Dale et al. (1999) and Fischl et al. (1999). [3]R Core Team (2018). [4]Brainard (1997), Kleiner et al. (2007), and Pelli (1997). [5]https://www.fil.ion.ucl.ac.uk/spm/software/spm8/. [6]https://doi.org/10.6084/m9.figshare.1344765.v24. [7]https://osf.io/w6dtz/. [8]https://osf.io/7jdgt/. [9]Oksanen et al. (2019). [10]Wickham (2016). [11]Wickham (2007). [12]Wickham (2011). [13]Widgren & Hulbert (2019). [14]Auguie (2019). [15]Venables & Ripley (2002). [16]Wolf (2019). [17]Maechler et al. (2019). pRF = Population receptive field. GLM = General linear model. cMDS = Classical (metric) multidimensional scaling. PTB = Psychtoolbox.

References

Auguie, B. (2019). egg: Extensions for ggplot2 [computer software]. URL: https://cran.r-project.org/package=egg.

Brainard, D. H. (1997). The Psychophysics Toolbox. *Spat. Vis.*, *10*, 433–436. doi:10.1163/156856897X00357. arXiv:arXiv:1011.1669v3.

Dale, A. M., Fischl, B., & Sereno, M. I. (1999). Cortical surface-based analysis - I. Segmentation and surface reconstruction. *Neuroimage*, *9*, 179–194. doi:10.1006/nimg.1998.0395.

Fischl, B., Sereno, M. I., & Dale, A. M. (1999). Cortical surface-based analysis: II. Inflation, flattening, and a surface-based coordinate system. *Neuroimage*, *9*, 195–207. doi:10.1006/nimg.1998.0396. arXiv:nimg.1998.0396.

Kleiner, M., Brainard, D. H., Pelli, D. G., Broussard, C., Wolf, T., & Niehorster, D. (2007). What's new in Psychtoolbox-3? [ECVP abstract supplement]. *Perception*, *36*. doi:http://journals.sagepub.com/doi/10.1177/03010066070360S101.

Maechler, M., Rousseeuw, P., Croux, C., Todorov, V., Ruckstuhl, A., Salibian-Barrera, M., Verbeke, T., Koller, M., Conceicao, E. L. T., & Of, M. A. d. P. (2019). robustbase: Basic robust statistics [computer software]. URL: http://cran.r-project.org/package=robustbase.

Oksanen, J., Blanchet, F. G., Friendly, M., Kindt, R., Legendre, P., McGlinn, D., Minchin, P. R., O'Hara, R. B., Simpson, G. L., Solymos, P., Stevens, M. H. H., Szoecs, E., & Wagner, H. (2019). Vegan: Community ecology package [Computer software]. URL: https://cran.r-project.org/package=vegan.

Pelli, D. G. (1997). The VideoToolbox software for visual psychophysics: Transforming numbers into movies. *Spat. Vis.*, *10*, 437–442. doi:10.1163/156856897X00366.

R Core Team (2018). R: A Language and environment for statistical computing [Computer software]. URL: https://www.r-project.org/.

Rousseeuw, P. J., & Croux, C. (1993). Alternatives to the median absolute deviation. *J. Am. Stat. Assoc.*, *88*, 1273–1283. doi:10.1080/01621459.1993.10476408.

Rousseeuw, P. J., Ruts, I., & Tukey, J. W. (1999). The bagplot: A bivariate boxplot. *Am. Stat.*, *53*, 382–387. doi:10.1080/00031305.1999.10474494.

Venables, W. N., & Ripley, B. D. (2002). *Modern applied statistics with S*. New York, NY: Springer. doi:10.1007/978-0-387-21706-2.

Wickham, H. (2007). Reshaping data with the reshape package. *J. Stat. Softw.*, *21*, 1–20. doi:10.18637/jss.v021.i12.

Wickham, H. (2011). The split-apply-combine strategy for data analysis. *J. Stat. Softw.*, *40*, 1–29. doi:10.18637/jss.v040.i01.

Wickham, H. (2016). *ggplot2: Elegant graphics for data analysis*. New York, NY: Springer. doi:10.1007/978-0-387-98141-3.

Widgren, S., & Hulbert, C. (2019). rmatio: Read and write 'Matlab' files [Computer software]. URL: https://cran.r-project.org/package=rmatio.

Wolf, H. (2019). aplpack : Another plot package [computer software]. URL: https://cran.r-project.org/package=aplpack.
